# Cost-consequence analysis of computer vision-based skin prick tests: implications for cost containment in Switzerland

**DOI:** 10.1186/s12913-024-11433-x

**Published:** 2024-08-26

**Authors:** Jean Pierre Uwitonze

**Affiliations:** 1 University of Bern, KPM Center for Public Management, Freiburgstr. 3, Bern, 3010 Switzerland; 2 Swiss Institute of Translational and Entrepreneurial Medicine, sitem-insel, Freiburgstr. 3, Bern, 3010 Switzerland

**Keywords:** Cost-consequence, Skin prick test, Hay fever, Cost savings, Sensitization, Allergy, Cost containment, Decision tree, Healthcare evaluation

## Abstract

**Background:**

Skin prick tests (SPTs), or intraepidermal tests, are often the first diagnostic approach for people with a suspected allergy. Together with the clinical history, SPTs allow doctors to draw conclusions on allergies based on the sensitization pattern. The purpose of this study is to investigate the potential cost consequences that would accrue to a Swiss University hospital after the adoption of computer vision-based SPTs.

**Methods:**

We conducted a cost-consequence analysis from a hospital’s perspective to evaluate the potential cost consequences of using a computer vision-based system to read SPT results. The patient population consisted of individuals who were referred to the allergology department of one of the five university hospitals in Switzerland, *Inselspital*, whose allergology department averages 100 SPTs a week. We developed an early cost-consequence model comparing two SPT techniques; computer vision*-*based SPTs conducted with the aid of Nexkin DSPT and standard fully manual SPTs. Probabilistic sensitivity analysis and additional univariate sensitivity analyses were used to account for uncertainty.

**Results:**

The difference in average cost between the two alternatives from a hospital’s perspective was estimated to be CHF 7 per SPT, in favour of the computer vison-based SPTs. *Monte Carlo* probabilistic simulation also indicated that SPTs conducted using the computer vision-based system were cost saving compared to standard fully manual SPTs. Sensitivity analyses additionally demonstrated the robustness of the base case result subject to plausible changes in all the input parameters, with parameters representing the costs associated with both SPT techniques having the largest influence on the incremental cost. However, higher sensitization prevalence rates seemed to favour the more accurate standard fully manual SPTs.

**Conclusion:**

Against the backdrop of rising healthcare costs especially in Switzerland, using computer-aided or (semi) automated diagnostic systems could play an important role in healthcare cost containment efforts. However, results should be taken with caution because of the uncertainty associated with the early nature of our analysis and the specific Swiss context adopted in this study.

**Supplementary Information:**

The online version contains supplementary material available at 10.1186/s12913-024-11433-x.

## Background

Skin prick tests (SPTs) and/or blood tests are often the first diagnostic approach for people with a suspected allergy [1]. In addition to being simple, safe and quick, SPTs are cheap and very reliable for diagnosing IgE-mediated allergic disease in patients with rhino conjunctivitis, asthma, urticaria, anaphylaxis, atopic eczema and suspected allergy [2]. SPTs provide evidence for sensitization and can help to confirm the diagnosis of a suspected type I allergy. Together with clinical history, SPTs allow to draw conclusions on allergies based on the sensitization pattern [1]. As such, SPT results only indicate sensitization and cannot be used as standalone allergy diagnostic methods [3].

Standard fully manual SPT procedure consists of putting drops of commercial extracts of food or pollen on the skin (usually forearm) which is then pierced with a small lancet, allowing the allergen to come into contact with the skin mast cells [4]. A positive reaction is shown by the typical “wheal and flare” reaction, which results from the erythema, pruritus and oedema that develop within 10 to 20 min. The SPT result is consequently given by the wheal diameter [2]. A wheal diameter of 3 mm or greater than the negative control is often arbitrarily considered a positive SPT result [1, 5]. The first SPTs were performed by the English physician Blackley in 1865 with the technique itself having evolved very little as it continues to be performed entirely manually today [6]. This standard manual SPT technique has several drawbacks that limit the utility of the tests, in many cases restricting them to a semi-quantitative assessment. Some of the drawbacks of standard SPTs include the possibility of making mistakes when reading the test results and more importantly, possible difficulties in the manual measurement of the reaction wheal sizes [7, 8].

Over the last 9 years, a team of engineers and allergologists in Spain have been working on the development of an computer vison-based (semi) automated system for reading SPT results which has led to the development of an electromedical device, Nexkin digital skin prick test (Nexkin DSPT), that helps automatically locate and measure wheal reactions. Nexkin DSPT captures a three-dimensional images of the forearm and using computer vision software, locates and measures the surface of the wheals caused by the allergens [9]. The device’s intended use is to aid in (semi) automated wheal detection and in the reading of the skin prick allergy test results. Nexkin DSPT’s computer vision-based technology therefore has the potential of addressing some of the shortcomings associated with standard fully manual SPTs [9, 10]. Nexkin DSPT also has the potential of impacting costs in hospitals and allergology units where thousands of SPTs are carried out yearly. However, its potential is yet to be substantiated through studies estimating the cost implications of computer vision-based SPTs. Besides a clinical study that showed less variability in wheal size results read with the aid of Nexkin DSPT in comparison to those read manually [10], no other study - to the best of our knowledge - has attempted to comparatively evaluate automated and standard manual SPTs nor quantify potential cost consequences of adopting computer vision-based SPT systems.

In the current context of growing concerns on rising healthcare costs and spending in Switzerland [11, 12], the objective of this study is to evaluate - from a Swiss university hospital’s perspective – potential cost consequences of adopting a computer vision-based SPT system in comparison to standard fully manual SPTs.

## Methods

### Study design

We conducted a cost-consequence analysis (CCA) and developed an early cost-consequence model to estimate potential cost consequences of using a computer vison-based SPT system (Nexkin DSPT) to read SPT results. Our cost-consequence analysis was limited to an “early” model because of data limitations and the novel nature of the Nexkin DSPT’s computer vision-based technology which made it more difficult to obtain robust parametrical inputs on a range of outcomes and costs necessary for a broader analysis [10, 13–15].

The early cost-consequence model was developed with the aid of a decision-tree that compared the expected cost values for both the computer vision-based SPT technique and the standard fully manual SPT technique [13, 16]. The patient population in our study consisted of individuals who were referred to the allergology department of one of the largest University hospitals in Switzerland, *Inselspital*, whose allergology department averages 100 SPTs a week. The costs were therefore determined from a healthcare provider’s perspective (hospital), with the costs accruing to the allergology department at *Inselspital*. All costs were presented in 2023 prices using Swiss francs (CHF). The computer vison-based SPT (Nexkin DSPT) device’s purchase cost was provided in Euros and an exchange rate of CHF 1 = €1 was assumed. The time horizon was taken to be a 1-year duration, with the assumption that 5,200 SPTs are carried out at *Inselspital* annually. Due to the short time horizon duration considered, we did not apply any form of discounting. The cost-consequence model was based on a decision analytical model constructed as a decision tree using the software program *TreeAge Pro 2023 v. R2.0*.

### Model structure

**Fig. 1:**
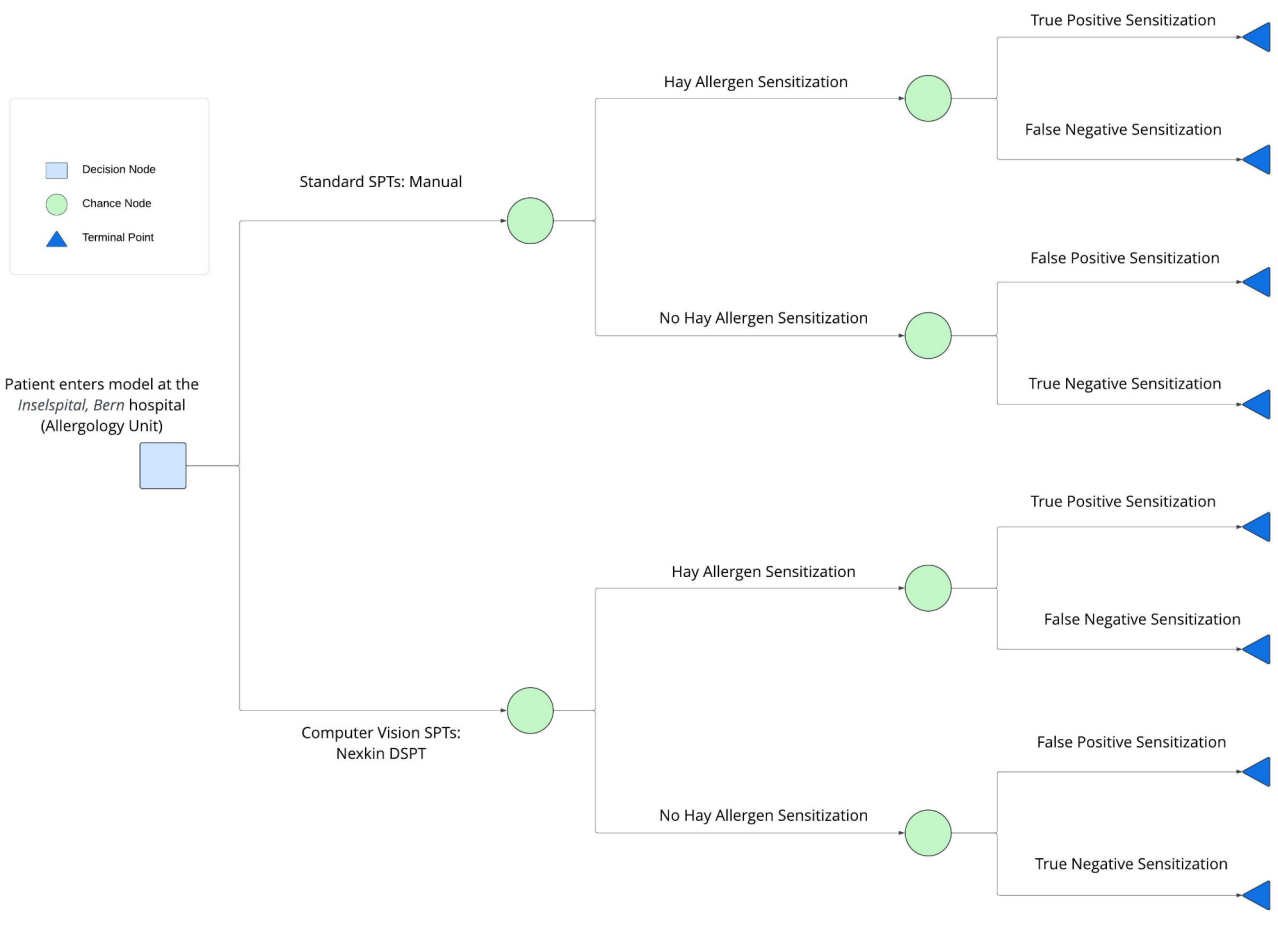
Decision tree schematic

The decision tree in Fig. 1 illustrates our modelled patient pathway and begins with a decision node that represents the two alternatives: standard fully manual SPTs and computer vision-based SPTs. Subsequent branches are denoted with a chance node, illustrated as a circle, and represent the possible events for patients referred to *Inselspital* with suspected sensitization. All possible events were based on *Inselspital’s* head allergology clinician’s experience.

The decision tree schematic therefore modelled the pathway through which a patient referred to the *Inselspital* hospital’s allergology department went through. Patients entered the model after referral by respective general practitioners (GPs) and left after SPTs were conducted by either of the two SPT techniques. Using the decision-tree model, we calculated the expected costs based on the summation of pathway values weighted by the pathway probabilities for each one of the SPT alternatives. We programmed the pathway probabilities as mutually exclusive sequences of events along the pathways illustrated in the decision tree schematic [13, 16].

Based on the *Inselspital’s* head allergology clinician’s experience, we assumed that a patient whose terminal node ended with a false positive sensitization result would have no direct cost consequence from a hospital’s perspective. However, we also reasonably assumed that patients with false negative terminal node outcomes would eventually get an additional referral to *Inselspital*, which would result in a repetition of SPT related costs for *Inselspital* because they would have to reconduct the SPT. Since our analysis was conducted from the hospital’s perspective, we considered “further tests” related costs solely accruing to the hospital. These costs were imputed for every terminal node with a false negative sensitization outcome only. This implies that we only considered cost implications potentially accruing to the *Inselspital’s* allergology department and not those potentially accruing to the patient. We also did not consider other costs potentially associated with alternative pathways beyond those illustrated in our decision tree.

### Model input parameters

Model parameters used in this study were obtained from two main sources, i.e., Literature (peer-reviewed literature, grey literature) and clinical expert input. Both the peer-reviewed and grey literature were retrieved from a pragmatic targeted literature search based on relevant key terms. Due to the limited number and quality of model inputs obtained from the targeted literature search, expert clinical input from the head of *Inselspital’s* allergology department and *Nexkin Medical* engineers was used to complement inputs retrieved from literature.

#### Pollen allergy (hay fever/pollinosis) in Switzerland

SPTs can be used to check for sensitization for multiple foods and inhalants [2], however, we chose to focus on pollen allergy SPTs since pollen allergy (hay fever/pollinosis) is the most common allergy in Switzerland [17]. While pollen allergy is a reaction to one or more types of pollen, the most common allergen positive sensitization is grass. In Switzerland, around 20% of the population suffers from a pollen allergy with the majority – 70% - sensitized to grass [17, 18]. For these reasons, our base case CCA considered sensitization for one of the most common types of pollen: timothy grass.

Hay fever (pollinosis) prevalence values were retrieved from the 1995 *prevalence of atopy and pollinosis in the adult population of Switzerland (SAPALDIA*^1^*) study* [19]. This is the only study whose results we could use as model inputs due to the robust data collection used in the study. Indeed, this study collected data from a random sample of 9,651 subjects aged from 18 to 60 years (mean age 41.1 years, males 49.2%), with complete allergy test data available for atopy, pollinosis and atopic asthma. The study reported results for 8,357 adults, with males accounting for 51.3% of the reported adult sample. Despite the old age of the study, the reference study is the largest and only study - to the best of our knowledge - to have ever collected country level data in the adult Swiss population with the aim of examining the relationship between environment and respiratory symptoms and diseases in Swiss adults. Recent studies discussing prevalence in Switzerland have neither been methodologically equal nor superior because they did not collect data from a representative sample of the entire Swiss population [20–22]. Furthermore, the mean age (41.1 years) and gender distribution (males 49.2%) reported in the 1995 SAPALDIA study [19] was confirmed by the head allergology clinician at *Inselspital* to be indicative of the mean age and gender proportions of the patient population referred to *Inselspital*.

The highest rate of sensitization in SPTs (adjusted wheal diameters 3 mm) observed in the SAPALDIA study was for grass pollen (total 12.7%, males 14.5%, females 10.9%; *p* < 0.001), followed by house dust mite (8.9%, no difference between males and females) and birch pollen (total 7.9%, males 7.9%, females 8%) [19]. Since we focus on timothy grass induced hay fever pollinosis in our study, we used sensitization values reported for grass pollen (Table 1).

#### Diagnostic inputs

We obtained diagnostic accuracy values (sensitivity and specificity) for standard manual SPTs in a study whose objective was to identify the best cut-off level for SPTs [23]. Despite the study concluding that a cut-off level of over 0 mm was best at identifying those with allergen specific IgE, we used 3 mm accuracy values since these reflect current standards and guidelines [1, 5] followed at the allergology department of *Inselspital*.

Diagnostic accuracy for automated SPTs on the other hand have been known to be inferior to manual SPTs that are widely accepted as the gold diagnostic standard for SPTs [7, 10]. Unpublished data from a clinical trial conducted in Spain alluded to lower accuracy for computer-vision based Nexkin DSPT [10]. Another study that discussed automated SPT techniques also suggested slightly inferior accuracy for computer vison based SPT systems [7]. This led us to systematically assuming and applying 2.5% lower accuracy values for Nexkin DSPT.

Visual illustrations and further details on how we obtained accuracy values (sensitivity & specificity) for the two SPT techniques can be found in the supplementary materials file. These values are also reported in Table 1.

**Table 1:** Hay fever (timothy grass) based diagnostic model inputs

Parameter	Count (*r*)*	Sample size (*n*)	EV**	Range	Distribution*** (*n*, *r*)	Source
Prevalence	1061	8357	0.127	[0.10–0.20]	*Beta (8357, 1061)*	[17–19]
Manual: Sensitivity	1485	2289	0.648	[0.64–0.66]	*Beta (1485.2289)*	[23]
Manual: Specificity	9366	9627	0.973	[0.97–0.98]	*Beta (9366*,* 9627)*	[23]
DSPT: Sensitivity	1448	2289	0.633	[0.62–0.64]	*Beta (1488*,* 2289)*	Assumption based on unpublished clinical trial study [10] data
DSPT: Specificity	9132	9627	0.948	[0.94–0.96]	*Beta (9132*,* 9627)*	Assumption based on unpublished clinical trial study [10] data

*Count denoted by *r* refers to the number of positives in the sample size denoted by *n*

**EV is the expected value or deterministic mean, which is a result of the division of counts, *r*, by sample size *n*

***Probabilistic distribution and associated uncertainty parameters *(n*,* r)* used to model the distributions in the software program *TreeAge Pro 2023 v. R2.0*

#### Costs

The costs (CHF) taken into consideration for this study -Table 2- reflect the costs associated with the two SPT techniques.

**Table 2:** Overview of cost related model inputs

Parameter	Value	Range (+-20%)	Distribution* (mean, SD)	Source
**Manual SPT**				
*Resource category 1*: Cost of materials	CHF 19.41			Communication with the head allergology clinician, *Inselspital*
*Resource category 2*: Human resources: Time per test	5.5 min		(5.5, 1.1)	Assumption based on unpublished clinical trial study [10] data
Human resources: Salary/employee costs per test	CHF 19.96		(19.96, 3.99)	[25], Communication with the head allergology clinician, *Inselspital*
***Total Manual***	**CHF 39.37**	**[31.5–47.2]**	***Gamma (39.37, 7.87)***	**-**
**Digital SPT**				
*Resource category 1*: Cost of materials	CHF 19.41			Communication with the head allergology clinician, *Inselspital*
*Resource category 2*: Human resources: Time/test	3 min		(3, 0.6)	Assumption based on unpublished clinical trial study [10] data
Human resources: Salary/employee costs per test	CHF 10.89		(1.089, 2.18)	[25], Communication with the head allergology clinician, *Inselspital*
*Resource category 3*: Cost of device per test (annual amortization & service fees)	CHF 0.40			Communication with *Nexkin Medical* engineers
***Total Digital SPT***	**CHF 30.70**	**[24.6–36.9]**	***Gamma (30.71,6.14)***	
***Further tests*** ^**^	**2**	**[1.6–2.4]**	***Gamma (2, 0.4)***	Communication with the head allergology clinician, *Inselspital*

*Probabilistic distribution and associated uncertainty parameters (mean, SD) used to model the distributions in the software program *TreeAge Pro 2023 v. R2.0*

** Further tests represent an additional test (total of 2 tests) that we imputed to patients whose decision tree pathway terminal node outcome was a false negative sensitization.

Only direct SPT related costs accruing to the hospital and were factored in the base case analysis. The first cost category associated with both SPT alternatives was the cost of materials (allergen extracts, lancets, etc.) which according to the head allergology clinician of *Inselspital*, costs CHF 19.41 on average per test for both alternatives. Both alternatives therefore had similar material costs, with the main difference coming from the amount of time spent reading and recording the SPT results and the resulting human resource costs.

Consequently, the second cost category was the human resource costs (salary/employee costs) associated with the health practitioner involved in the SPT procedure. Since both the manual and the computer vision-based SPTs require a 15-min wait time for the allergen reaction to appear [24], the time considered in our cost calculations was 5.5 min and 3 min for the standard fully manual SPT computer vision-based Nexkin DSPT respectively. This is indeed the respective amount of time spent reading and recording the results after the 15-min wait. Time estimates were informed by unpublished clinical trial data used for a previous study that compared the variability of wheal size readings conducted by both SPT techniques [10]. We did not calculate human resource costs for the 15-min wait since nurses could reasonably multitask while waiting for the 15 min to elapse. Based on an average annual *Inselspital* nurse salary of CHF109,743.3, a 52-year week and a 42-hour contract, the average human resources component of the manual SPT and Nexkin DSPT was established at CHF 19.96 and CHF 10.89 respectively [25].

Since the Nexkin DSPT device is an asset, we also included depreciation costs which refers to the amount of capital that is ‘used-up’ in one year [26]. The simplest approach to accounting for depreciation is known as *straight-line depreciation* which assumes that the services from the capital item are evenly distributed over the useful life of the capital item [27]. *NexkinMedical *engineers suggested a 10-year useful life period for the Nexkin DSPT ^®^ device, and a capital value of CHF 10,400. The annual depreciation amounted to CHF 1,040 on top of which an annual service cost of CHF 1,040 was added. We assumed that an average of 5,200 SPTs were conducted every year at *Inselspital’s* allergology department, which led to the addition of an extra annual cost of CHF 0.4 per computer vison-based SPT *(*Table 2*).*

#### Further tests

As alluded to earlier, a false negative sensitization terminal node outcome could lead to “further tests” at *Inselspital* because of a second GP referral for a patient who experienced uncomfortable symptoms after a false negative sensitization outcome. Unlike false negative SPT results, patients with false positive outcomes are unlikely to undergo further tests because SPTs are not standalone diagnostic procedures [9] and need to be coupled with the patient’s clinical history for allergy diagnosis. We therefore assumed that “further tests” would lead to a repetition of the SPT related costs for the hospital, and only for patients with false negative sensitization outcomes. The parameters in the last row of Table 2 accounted for this by assuming false negative terminal node outcomes would lead to an average of two SPTs for concerned patients.

### Decision rule

After assigning costs and probabilities to each corresponding event, the product of the costs and their matching probabilities was summed across the nodes for each branch of the decision tree in Fig. 1 to approximate the average cost of the branches for each of the two SPT alternatives [13, 16]. The incremental cost was estimated by finding the difference in expected average costs incurred when performing the SPTs manually and the expected average costs incurred when SPTs were performed with the aid of the computer vison-based system, Nexkin DSPT.

### Sensitivity analysis

To check the robustness of the base case results, deterministic univariate sensitivity analyses were conducted. Analyses were performed by varying one parameter at a time within a plausible range while keeping the others constant, thereby estimating the impact of each parameter on the incremental cost [28].

The plausible ranges for all parameters reflected the range of uncertainty associated with our model input parameters and are illustrated in the “ranges” columns of Tables 1 and 2. The lower bound prevalence value of 0.10 was the lowest prevalence rate for timothy grass induced hay fever [19] recorded in our reference study while the upper bound 0.20 represents current Swiss hay fever prevalence rate among Swiss adults [17]. The ranges for sensitivity and specificity values on the other hand were based on the 95% confidence intervals (CI) provided in our reference study for SPT accuracy [23]. Cost parameter ranges were based on a 20% +- variation in prices observed by the head clinician of the allergology department at *Inselspital*. Finally, the number of further tests ranged from 1.6 to 2.4, which is also a +-20% range.

#### Probabilistic sensitivity analysis (PSA)

To account for the overall parameter uncertainty in our model, we conducted probability sensitivity analysis (PSA) by assigning probability distributions to each input parameter as depicted in the “distribution” column of both Tables 1 and 2. Cost parameters were assigned *Gamma* distributions while probability parameters were assigned *Beta* distributions [29]. We conducted PSA by drawing *Monte Carlo* simulations from the probability distributions. The probabilistic model simulations stabilised at approximately 10,000 *Monte Carlo* simulations which yielded 10,000 result estimates.

## Results

### Cost-consequence model

**Fig. 2:**
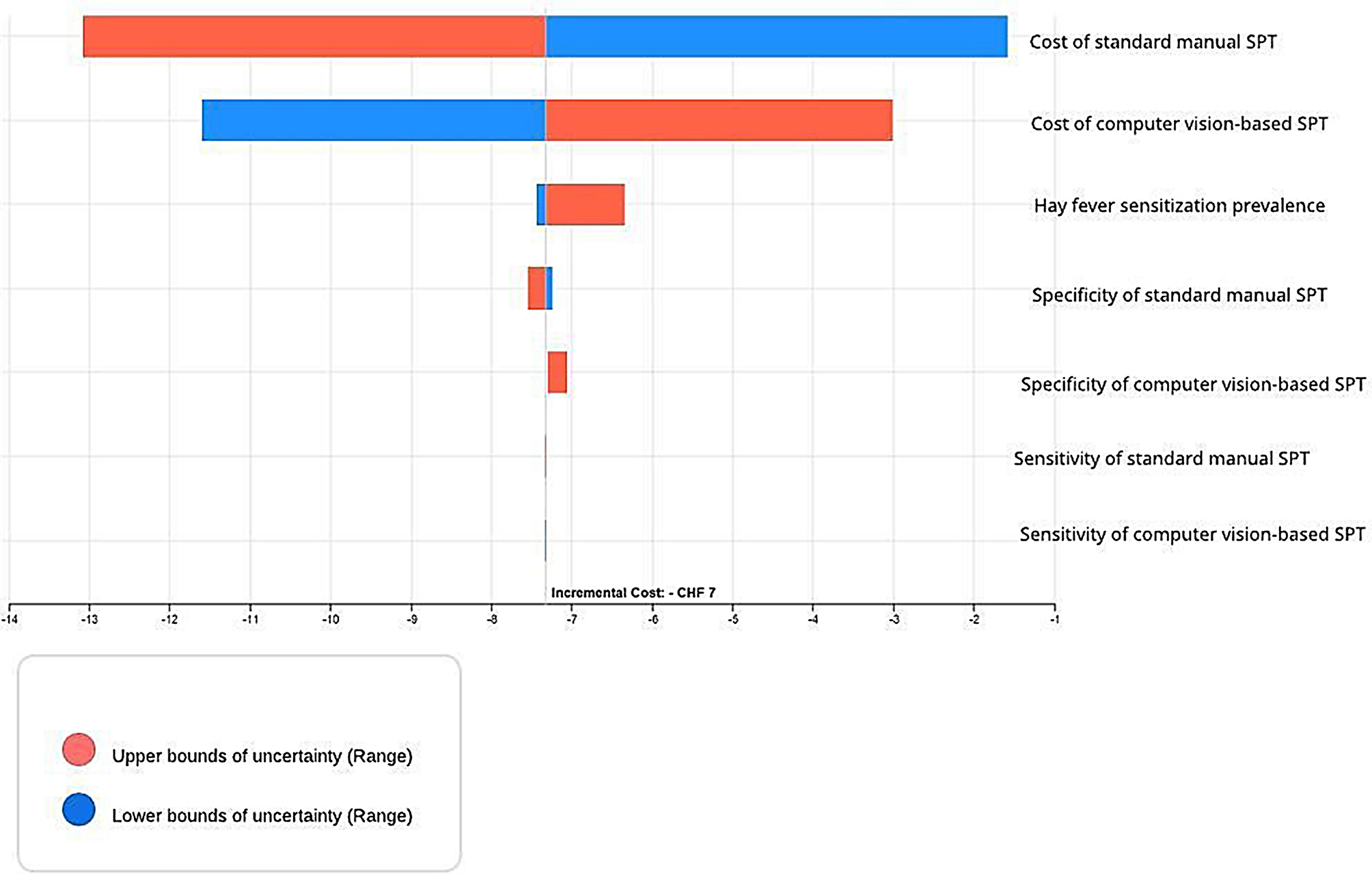
Incremental active payoff tornado chart of base case analysis

From the perspective of a Swiss university hospital’s (*Inselspital*) allergology department, the average expected value of costs saved by performing SPTs on patients using a computer vision-based system was approximately CHF 7 per test. This result is illustrated in the incremental active payoff tornado chart in Fig. 2.

### Sensitivity analysis

Tornado diagrams show the extent to which uncertainty in individual parameters affect the incremental cost as the various input parameters vary within the ranges provided in Tables 1 and 2 [30]. Results from the deterministic tornado diagram shown in Fig. 2 showed that all the parameters remained within a negative incremental cost, thus demonstrating robustness of the base case result to plausible changes in all the input parameters. Blue bars show the impact of low values of the parameter on the incremental cost of using computer vision-based Nexkin DSPT as opposed to using standard fully manual SPT procedures. Red bars on the other hand show the impact of high values of the respective parameters on the incremental cost.

Parameters representing the costs associated with the two SPT alternatives were the most influential because changes in these two parameters had the largest influence on the incremental cost. As expected, higher costs of fully manual (computer vision-based Nexkin DSPT) SPTs reduced (increased) incremental costs of conducting computer vison-based SPTs while lower costs of manual (Nexkin DSPT) SPTs increased (reduced) incremental costs.

Higher hay fever sensitization prevalence also favoured standard fully manual SPTs because higher hay fever sensitization prevalence levels increased incremental costs of conducting computer vison-based SPTs. A potential explanation behind this observation would be the lower accuracy (sensitivity and specificity values) associated with the computer vison-based SPT techniques [7]. In fact, higher allergen prevalence rates would result in a higher proportion of diagnostic inaccuracies for computer vison-based SPTs and potentially lead to worse cost consequences.

### Probability sensitivity analysis (PSA)

**Fig. 3:**
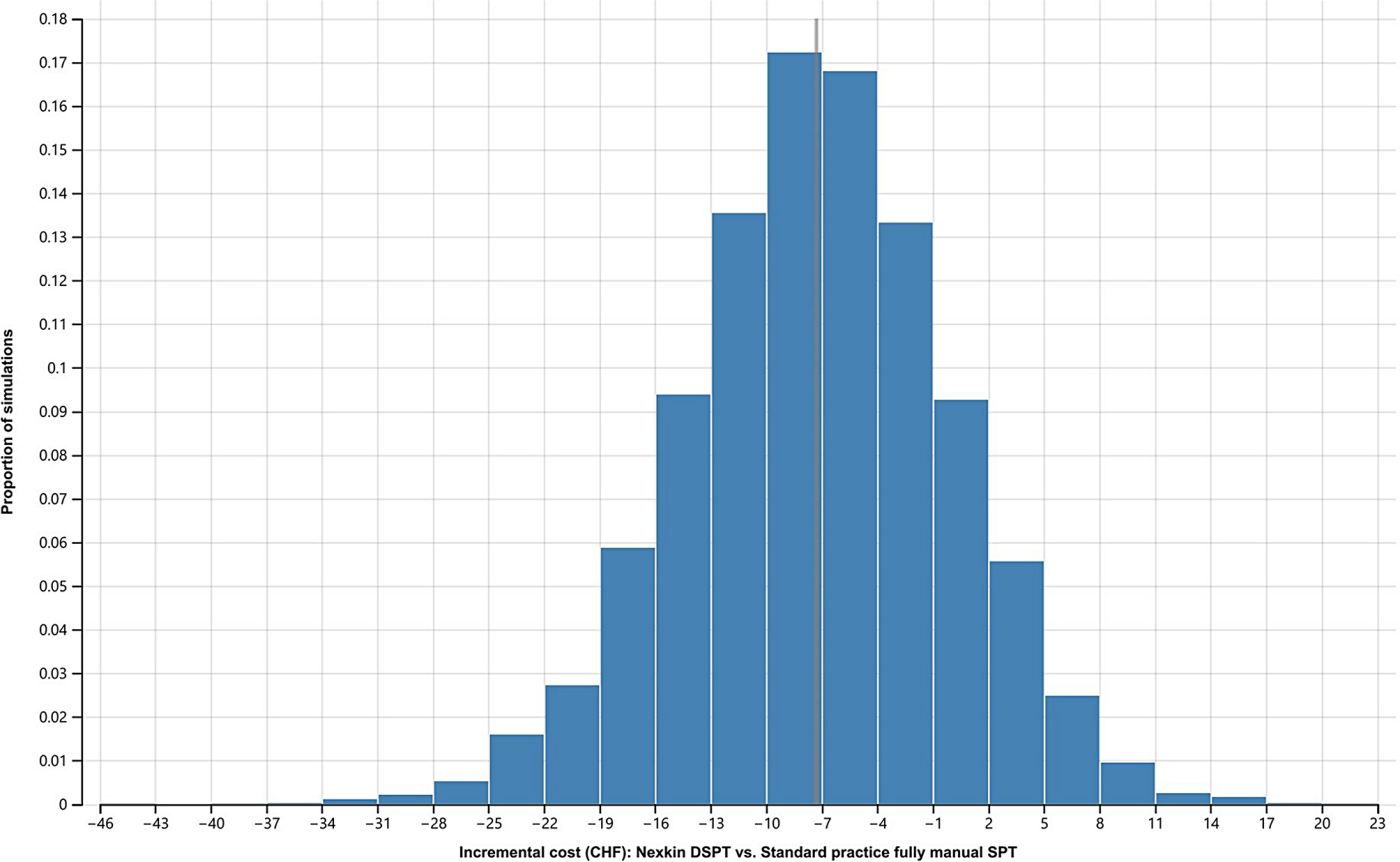
Monte Carlo simulation distribution of incremental cost

As illustrated in Fig. 3, our *Monte Carlo* simulation which accounted for general uncertainty around our model input parameters also indicated that SPTs conducted using the computer vison-based SPT system were cost-saving compared to standard fully manual SPTs in the majority of the simulations, i.e., incremental cost of conducting manual SPTs in comparison to computer vison-based SPTs was close to the base case result of CHF 7 for most of the simulations. The simulations were normally distributed.

## Discussion

### Findings

This study showed that performing SPTs using a computer vision-based system (Nexkin DSPT) in the allergology department of a Swiss university hospital results in average savings of CHF 7 per test compared to fully manual standard SPT techniques. Deterministic sensitivity analyses confirmed our model’s robustness because computer vision-based SPTs remained cost-saving regardless of plausible changes in input parameters. The robustness of the findings was further investigated using probabilistic sensitivity analysis (PSA) which demonstrated that computer vison-based SPTs were cost saving with a high degree of certainty. However, the computer vison-based system became less cost saving as; (i). the costs associated with the standard fully manual SPT declined and (ii) hay fever sensitization prevalence rates increased. Higher allergen prevalence rates would therefore favour the more accurate manual SPTs, a result in line with what some studies have shown regarding the importance of accuracy in minimising costs [31, 32].

We developed an early cost-consequence model and as such, our results provide an initial understanding of some of the costs that could be saved by using (semi) automated devices in diagnostic procedures. Our observations are particularly relevant in Switzerland where healthcare costs have been incessantly rising and where cost containment remains a prime concern for all major stakeholders of the Swiss healthcare system, including hospitals [11].

### Comparison to existing literature

A recent study showed that the Nexkin DSPT device provided more consistent readings in comparison to standard manual SPTs [10]. However, this study focused on wheal size reading consistency and did not address its cost saving potential. Another study pointed out the need for the development of an automated system of conducting prick tests and duly spelled out the optimality and effectiveness of automated SPTs in terms of time and materials [9]. Again, this study did not quantify the cost saving potential of automated SPTs vs. standard manual SPT procedures. To the best of our knowledge no other studies have evaluated the potential cost savings associated with the use of (semi) automated devices to perform SPTs at a hospital. Our study therefore takes an important step forward in the advancement of the adoption of (semi) automated diagnostic techniques which if adopted at scale, could play a key role in healthcare cost containment efforts.

### Limitations

Data limitations and the novel nature of the computer vison-based SPT technique precluded us from conducting a more elaborate and comprehensive cost effectiveness analysis (CEA) with a broader perspective that would have not only considered cost implications but also other important health outcomes [13, 16]. In this context, a comprehensive CEA would have been premature for this study thus making a cost-consequence analysis (CCA) ideal for the purposes of our study.

CCAs have the advantage of allowing cost and outcome disaggregation which affords readers and decision makers the possibility of forming their own opinion on the relevance and relative importance to their decision-making context [13, 14, 33] Furthermore, CCAs are a straightforward form of evaluation for new health technologies and interventions with the potential of having important consequences whose relevance may differ depending on perspective (healthcare, patient, etc.) [33, 34]. For these reasons, we developed an early cost-consequence model and evaluated the cost consequences solely from a hospital’s perspective.

Despite these advantages, CCAs have been criticised for their limited generalizability due to their context specific nature and subjective importance of cost components which vary depending on decision makers primary concerns [14]. Concerns have also been raised regarding transparency of costs and outcomes used in CCAs which may lead to cherry picking positive results [15]. The use of CCA may therefore present a limitation to the current study. Moreover, we did not develop an ideal CCA which similar to comprehensive CEAs ideally consider all key health/cost related outcomes in addition to analysing results from multiple perspectives [14, 33].

For instance, our early cost-consequence model was analysed from a hospital’s perspective which implies that we did not consider important health/cost outcomes that would have helped provide a more informed decision from a broader societal perspective. Notable omissions include eventual GP related costs and patient related quality of life outcomes for patients requiring further consultations after a false negative sensitization outcome in our modelled patient pathway. The limited (hospital only) evaluation perspective and early nature of our model also precluded us from considering, among other potential patient related costs, prescription costs for medication and/or complementary tests prescribed to patients with both true and false positive sensitization outcomes in our modelled patient pathway. Our failure to consider such costs and outcomes ignores the broader societal implications of computer vision-based SPTs and as a result, limits the generalizability of our results.

Additionally, it would be wrong to assume that the lower diagnostic accuracy values associated with the computer vison-based SPT device would have been without effect on health outcomes, had our analysis considered health outcomes. Studies exploring the impact of diagnostic accuracy on health outcomes and healthcare costs have shown that inferior or delayed diagnostic accuracy increased health risks, worsened patient outcomes and increased healthcare costs due to unnecessary interventions [31, 32]. Nexkin DSPT’s lower diagnostic accuracy would have most probably had a negative impact or at least lowered the cost savings demonstrated in this study.

It is therefore important to note that while we achieve our objective, our early cost-consequence model does not capture the entire spectrum of cost and health outcome consequences that could have been captured had we considered patient related (health) outcomes and other broader consequences. Arguably low-quality data constantly used in our analysis also adversely affects the robustness of the model. First, hay fever prevalence values in Switzerland we used are based on a study that dates back to 1995 [19]. Input values sourced from the study might therefore be considered as outdated, despite the head allergology clinician at the *Inselspital* hospital confirming similar observations in their allergology department’s day to day. Second, the use of non-empirical parameter estimates and assumptions based on clinical expertise potentially weakens our conclusions because it could result in over and/or underestimations. Our context specific assumptions could also limit the transferability of our findings to other healthcare institutions and health systems where the suspected allergy patients’ care pathway differs from that of patients referred to a Swiss university hospital.

### Recommendations (future studies)

Considering the limitations discussed and the early nature of our analysis, it would be suitable to collect more robust model input data, particularly data on health outcomes in order to further the evidence our study generated. The collection of data would indeed facilitate a broader evaluation perspective integrating the view of all key societal player (patients, hospital, and other payer institutions) thus providing a better understanding of the entire spectrum of the consequences of computer vision-based systems in conducting SPTs. To this end, we are currently conducting a clinical trial at *Inselspital* whose data collection objectives include furthering the early CCA model developed in this study^2^.

Future evaluation models should also consider extending the analysis to multiple healthcare settings.

## Conclusion

Our study is the first of its kind to evaluate the potential cost savings associated with the use of (semi) automated devices to perform SPTs at a hospital. Despite our study’s various limitations that restricted our analysis to an early cost-consequence model, we achieve our objective and conclude that the use of computer vison-based SPTs has the potential of saving CHF 7 per test on average in comparison to standard fully manual SPTs. Against the backdrop of rising healthcare costs, especially in Switzerland, using similar computer vison-based diagnostic systems could contribute in cost containment efforts. By developing an early cost-consequence model that evaluates the consequences of adopting (semi) automated technologies in the care pathway, we also generate early evidence in healthcare economics and healthcare policy that future research can build on. Ultimately, our study contributes to literature exploring cost implications of digital technology adoption in healthcare institutions. However, results should be taken with caution due to the early nature of our model and the Swiss hospital specific setting in which our analysis was conducted.

### Electronic supplementary material

Below is the link to the electronic supplementary material.

Supplementary Material 1

## Data Availability

The data referenced and analysed in the context of the present study are available from the sources indicated by author.

## Footnotes

1 Swiss study on air pollution and lung diseases in adults.

2 In collaboration with *Nexkin Medical* and *Inselspital Bern*, a new clinical trial study protocol comparing the accuracy (sensitivity & specificity) and time required for both SPT techniques was recently approved by the relevant Swiss authorities.

## Abbreviations

CHF: Swiss Francs
CCA: Cost-consequence analysis
CEA: Cost-effectiveness analysis
GP: General practitioner
SPT: Skin prick test

## References

[1] Heinzerling L, Mari A, Bergmann K-C, Bresciani M, Burbach G, Darsow U, et al. The skin prick test – European standards. Clin Transl Allergy. 2013;3:3. 10.1186/2045-7022-3-3. 23369181 10.1186/2045-7022-3-3 PMC3565910

[2] Gomes-Belo J, Hannachi F, Swan K, Santos AF. Advances in Food Allergy diagnosis. CPR. 2018;14:139–49. 10.2174/1573396314666180423105842. 10.2174/1573396314666180423105842 29692253

[3] Kattan JD, Sicherer SH. Optimizing the diagnosis of food allergy. Immunol Allergy Clin North Am. 2015;35:61–76. 10.1016/j.iac.2014.09.009. 25459577 10.1016/j.iac.2014.09.009 PMC4644667

[4] Bernstein IL, Storms WW. Practice parameters for allergy diagnostic testing. Joint Task Force on Practice parameters for the diagnosis and Treatment of Asthma.The American Academy of Allergy, Asthma and Immunology and the American College of Allergy, Asthma and Immunology. Ann Allergy Asthma Immunol. 1995;75(6 Pt 2):543–625. https://pubmed.ncbi.nlm.nih.gov/7494078/. 8521115

[5] Boyce JA, Assa’ad A, Burks AW, Jones SM, Sampson HA, Wood RA, et al. Guidelines for the diagnosis and management of food allergy in the United States: report of the NIAID-sponsored expert panel. J Allergy Clin Immunol. 2010;126:S1–58. 10.1016/j.jaci.2010.10.007. 21134576 10.1016/j.jaci.2010.10.007 PMC4241964

[6] Blackley C. Experimental researches on the causes & nature of catarrhus Aestivus. 1st ed. Baillière Tindall & Cox; 1873. https://www.ncbi.nlm.nih.gov/pmc/articles/PMC1293079/.

[7] Justo X, Diaz I, Gil JJ, Gastaminza G. Medical device for Automated Prick Test Reading. IEEE J Biomed Health Inf. 2018;2018:895–903. 10.1109/JBHI.2017.2680840. 10.1109/JBHI.2017.2680840 28362597

[8] Ansotegui et al. IgE allergy diagnostics and other relevant tests in allergy, a World Allergy Organization position paper. World Allergy Organization Journal. 2020;2020. 10.1016/j.waojou.2019.100080 10.1016/j.waojou.2019.100080 PMC7044795 32128023

[9] Justo X, Díaz I, Gil JJ, Gastaminza G. Prick test: evolution towards automated reading. Allergy. 2016;71:1095–102. 10.1111/all.12921. 27100940 10.1111/all.12921

[10] La MdP M-P, Núñez-Córdoba JM, Tejero E, Matellanes Ó, Quan PL, Carvallo Á, et al. Reliability of a novel electro-medical device for wheal size measurement in allergy skin testing: an exploratory clinical trial. Allergy. 2023;78:299–301. 10.1111/all.15474. 35950712 10.1111/all.15474 PMC10087902

[11] Braendle T, Colombier C. Budgetary targets as cost-containment measure in the Swiss healthcare system? Lessons from abroad. Health Policy. 2020;124:605–14. 10.1016/j.healthpol.2020.05.007. 32473748 10.1016/j.healthpol.2020.05.007 PMC7255250

[12] Stucki M, Schärer X, Trottmann M, Scholz-Odermatt S, Wieser S. What drives health care spending in Switzerland? Findings from a decomposition by disease, health service, sex, and age. BMC Health Serv Res. 2023;23:1149. 10.1186/s12913-023-10124-3. 37880733 10.1186/s12913-023-10124-3 PMC10598929

[13] Drummond M. Methods for the economic evaluation of health care programmes. New York NY USA: Oxford United Kingdom; 2015. https://books.google.co.uk/books?id=lvWACgAAQBAJ.

[14] Mauskopf JA, Paul JE, Grant DM, Stergachis A. The role of cost-consequence analysis in healthcare decision-making. PharmacoEconomics. 1998;13:277–88. 10.2165/00019053-199813030-00002. 10178653 10.2165/00019053-199813030-00002

[15] GOV.UK. Cost consequence analysis: health economic studies. 2020. https://www.gov.uk/guidance/cost-consequence-analysis-health-economic-studies#more-information-and-resources. Accessed 23 May 2024.

[16] Briggs AH, Claxton K, Sculpher MJ. Decision modelling for health economic evaluation. Oxford: Oxford University Press; 2006. https://pure.york.ac.uk/portal/en/publications/decision-modelling-for-health-economic-evaluation.

[17] Allergiezentrum Schweiz. aha!. (11. 09 2023). Pollen allergy (hay fever). https://www.aha.ch/swiss-allergy-centre/allergies-intolerances/pollen-allergy/pollen-allergy-hay-fever (2023).

[18] Ballmer-Weber B, Helbling A. Allergische Rhinitis. Swiss Med Forum. 2017. 10.4414/smf.2017.02897. 10.4414/smf.2017.02897

[19] Wüthrich B, Schindler C, Leuenberger P, Ackermann-Liebrich U. Prevalence of atopy and pollinosis in the adult population of Switzerland (SAPALDIA study). Swiss study on Air Pollution and Lung diseases in adults. Int Arch Allergy Immunol. 1995;106:149–56. 10.1159/000236836. 7819743 10.1159/000236836

[20] SAPALDIA. Methods and participation in the cross-sectional part of the Swiss study on Air Pollution and Lung Diseases in Adults. 10.1007/BF01318136 9151378

[21] Braun-Fahrländer C, Gassner M, Grize L, Takken-Sahli K, Neu U, Stricker T, et al. No further increase in asthma, hay fever and atopic sensitisation in adolescents living in Switzerland. Eur Respir J. 2004;23:407–13. 10.1183/09031936.04.00074004. 15065830 10.1183/09031936.04.00074004

[22] Frei T, Gassner E. Trends in prevalence of allergic rhinitis and correlation with pollen counts in Switzerland. Int J Biometeorol. 2008;52:841–7. 10.1007/s00484-008-0178-z. 18751737 10.1007/s00484-008-0178-z

[23] Bousquet P-J, Chatzi L, Jarvis D, Burney P. Assessing skin prick tests reliability in ECRHS-I. Allergy. 2008;63:341–6. 10.1111/j.1398-9995.2007.01581.x. 18070229 10.1111/j.1398-9995.2007.01581.x

[24] Bousquet, et al. Practical guide to skin prick tests in allergy to aeroallergens, position paper. Allergy. 2012;2012:18–24. 10.1111/j.1398-9995.2011.02728.x. 10.1111/j.1398-9995.2011.02728.x 22050279

[25] Statistik BF. Salaire mensuel brut (valeur centrale) pour une sélection de professions médicales selon les années de service et le sexe – 2018 | Tabelle | Bundesamt für Statistik. 30/09/2021.

[26] Walker D, Kumaranayake L. Allowing for differential timing in cost analyses: discounting and annualization. Health Policy Plan. 2002;17(1):112-8. 10.1093/heapol/17.1.112. PMID: 11861593. 10.1093/heapol/17.1.112 11861593

[27] Kumaranayake L. The real and the nominal? Making inflationary adjustments to cost and other economic data. Health Policy Plann. 2000;15:230–4. 10.1093/heapol/15.2.230. 10.1093/heapol/15.2.230 10837047

[28] Fox-Rushby J, Cairns J. Economic evaluation. Berkshire: Open University Press;https://books.google.be/books?id=tczsAAAAQBAJ&printsec=frontcover&hl=de&source=gbs_ge_summary_r&cad=0#v=onepage&q&f=false(2010).

[29] Briggs A. Probabilistic analysis of cost-effectiveness models: statistical representation of parameter uncertainty. Value Health. 2005;8:1–2. 10.1111/j.1524-4733.2005.08101.x. 15841888 10.1111/j.1524-4733.2005.08101.x

[30] McCabe C, Paulden M, Awotwe I, Sutton A, Hall P. One-way sensitivity analysis for probabilistic cost-effectiveness analysis: conditional expected incremental net benefit. PharmacoEconomics. 2020;38:135–41. 10.1007/s40273-019-00869-3. 31840216 10.1007/s40273-019-00869-3 PMC6977148

[31] Dave N, Bui S, Morgan C, Hickey S, Paul CL. Interventions targeted at reducing diagnostic error: systematic review. BMJ Qual Saf. 2022;31:297–307. 10.1136/bmjqs-2020-012704. 34408064 10.1136/bmjqs-2020-012704

[32] Koffijberg H, van Zaane B, Moons KGM. From accuracy to patient outcome and cost-effectiveness evaluations of diagnostic tests and biomarkers: an exemplary modelling study. BMC Med Res Methodol. 2013;13:12. 10.1186/1471-2288-13-12. 23368927 10.1186/1471-2288-13-12 PMC3724486

[33] Gage H, Kaye J, Owen C, Trend P, Wade D. Evaluating rehabilitation using cost-consequences analysis: an example in Parkinson’s disease. Clin Rehabil. 2006;20:232–8. 10.1191/0269215506cr936oa. 16634342 10.1191/0269215506cr936oa

[34] Coast J. Is economic evaluation in touch with society’s health values? BMJ. 2004;329:1233–6. 10.1136/bmj.329.7476.1233. 15550430 10.1136/bmj.329.7476.1233 PMC529373

