# Supplementary material for: Cost-consequence analysis of computer vision-based skin prick tests: implications for cost containment in Switzerland
Source: BMC Health Serv Res. 2024 Aug 26;24:988. doi: 10.1186/s12913-024-11433-x (PMC11348610; doi:10.1186/s12913-024-11433-x)
Supplement: Supplementary file 1 — Supplementary Material 1 [file 12913_2024_11433_MOESM1_ESM.docx]

**Model input parameters for computer vision-based Nexkin DSPT**

Unpublished clinical trial data from a study conducted in Spain alluded to lower accuracy (sensitivity & specificity) for computer-vision based Nexkin DSPT [1]. The initial objectives of this Nexkin DSPT study included the estimation of sensitivity and specificity values for Nexkin DSPT as illustrated on *Figure 2*. However, limitations encountered during the study limited the study’s objectives to the comparison of the variations in wheal reaction size measurements and as a result, data and results on Nexkin DSPT’s accuracy were not published. Based on the unpublished clinical trial data and another study that suggested lower accuracy for automated SPT techniques [2], we systematically applied an average of 2.5% lower accuracy values for Nexkin DSPT in comparison to values retrieved and for fully manual DSPTs [3]. All accuracy values and input parameters for both SPT techniques are illustrated in *Table 1* of our main article.


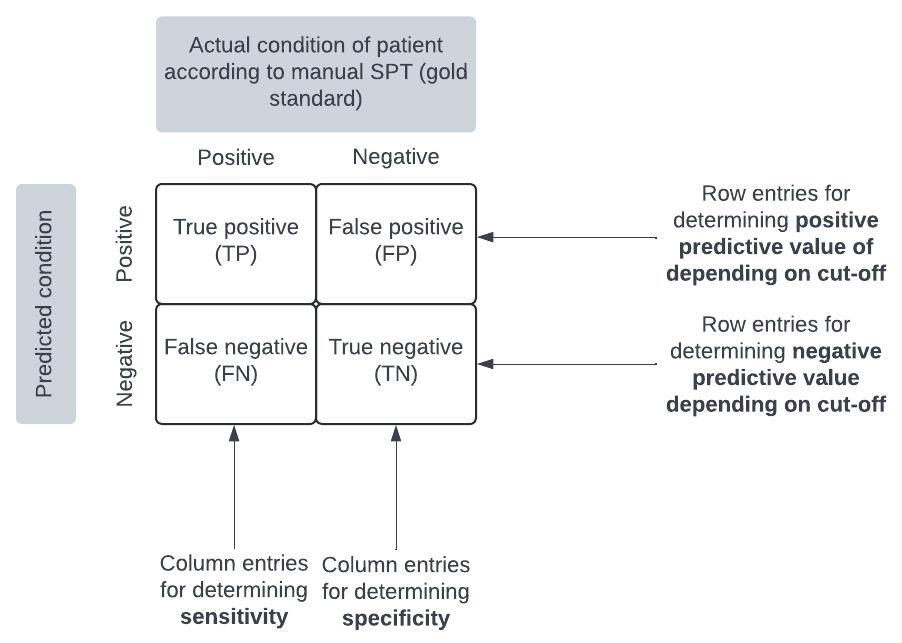


Figure 1: Estimation of standard SPT accuracy (specificity and sensitivity) values


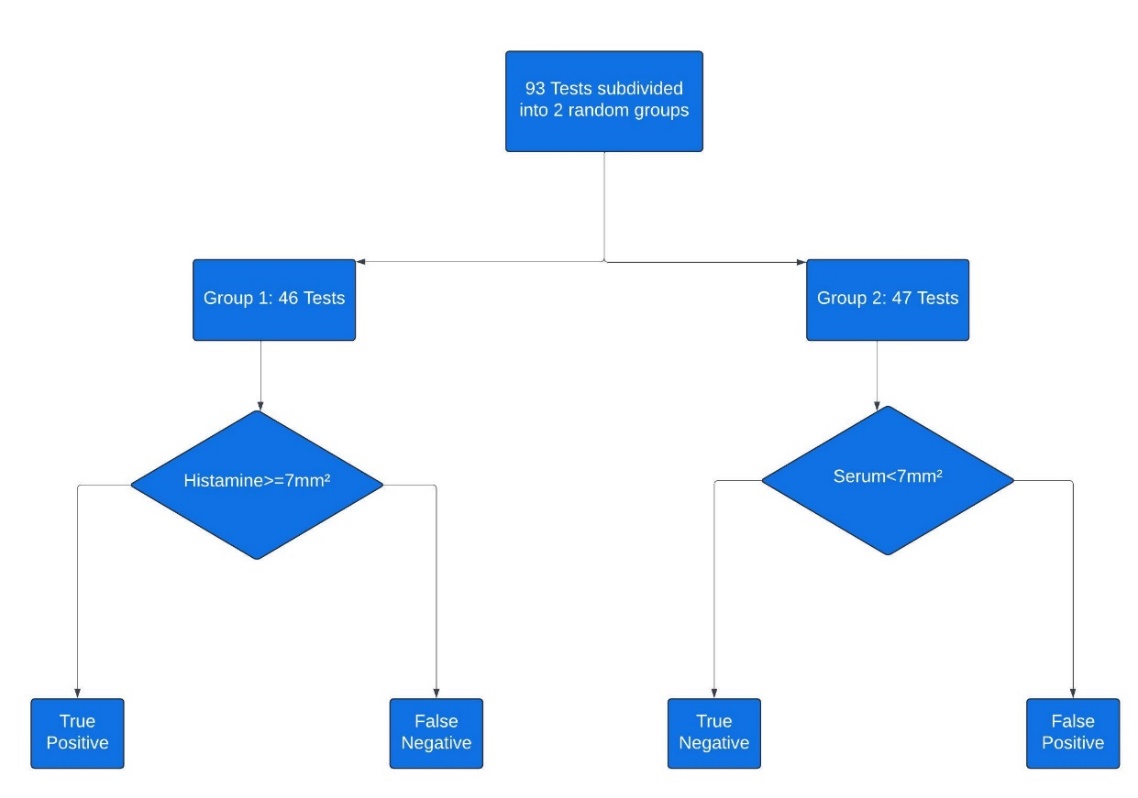


Figure 2: Estimation computer vision-based Nexkin DSPT (sensitivity and specificity)values

**References**

1. La Morales-Palacios MdP, Núñez-Córdoba JM, Tejero E, Matellanes Ó, Quan PL, Carvallo Á, et al. Reliability of a novel electro-medical device for wheal size measurement in allergy skin testing: An exploratory clinical trial. Allergy. 2023;78:299–301. doi:10.1111/all.15474.

2. Justo X, Diaz I, Gil JJ, Gastaminza G. Medical Device for Automated Prick Test Reading. IEEE J Biomed Health Inform. 2018;2018:895–903. doi: 10.1109/JBHI.2017.2680840

3. Bousquet P-J, Chatzi L, Jarvis D, Burney P. Assessing skin prick tests reliability in ECRHS-I. Allergy. 2008;63:341–6. doi:10.1111/j.1398-9995.2007.01581.x.
